# Design, Synthesis and Bioactivity Evaluation of Novel β-carboline 1,3,4-oxadiazole Derivatives

**DOI:** 10.3390/molecules22111811

**Published:** 2017-10-29

**Authors:** Zhi-Jun Zhang, Jing-Jing Zhang, Zhi-Yan Jiang, Guo-Hua Zhong

**Affiliations:** 1 Key Laboratory of Natural Pesticide and Chemical Biology, Ministry of Education, Guangzhou 510642, China; zhangzhijun198803@163.com (Z.-J.Z.); zhangjingjing@stu.scau.edu.cn (J.-J.Z.); zyjiang@stu.scau.edu.cn (Z.-Y.J.); 2 Lab of Insect Toxicology, South China Agricultural University, Guangzhou 510642, China

**Keywords:** β-carboline, cytotoxic activity, Sf9 cells, *Spodoptera litura*, growth inhibitory activity

## Abstract

A series of novel β-carboline 1,3,4-oxadiazole derivatives were designed and synthesized, and the in vitro cytotoxic activity against Sf9 cells and growth inhibitory activity against *Spodoptera litura* were evaluated. Bioassay results showed that most of these compounds exhibited excellent in vitro cytotoxic activity. Especially, compound **37** displayed the best efficacy in vitro (IC_50_ = 3.93 μM), and was five-fold more potent than camptothecin (CPT) (IC_50_ = 18.95 μM). Moreover, compounds **5** and **37** could induce cell apoptosis and cell cycle arrest and stimulate Sf-caspase-1 activation in Sf9 cells. In vivo bioassay also demonstrated that compounds **5** and **37** could significantly inhibit larvae growth of *S. litura* with decreasing the weight of larvae and pupae. Based on these bioassay results, compounds **5** and **37** emerged as lead compounds for the development of potential insect growth inhibitions.

## 1. Introduction

**Figure 1:**
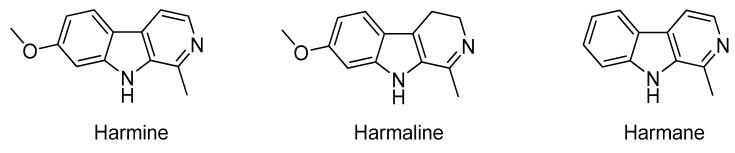
Structure of the major β-carboline alkaloids of *P. harmala*.

In recent years, potential impact of synthetic pesticides on the environment and human health has been of great concern, which highlights the need for environmentally friendly pesticides to protect crops from insect infestation. Accordingly, plant-derived extracts and their bioactive natural compounds have been considered as biorational alternatives with structural diversity and complexity, biodegradability, environmental protection, pest specificity and reduced mammalian toxicity to replace synthetic agrochemicals for pest management [1]. Especially, further modification and structural optimization of novel insecticidal leads from plant origin have recently been important methods for the research and development of new pesticides [2,3,4,5]. Harmine and its analogs, such as harmaline and harmane (Figure 1), belonging to the β-carboline alkaloids, were isolated from *Peganum harmala* and *Eurycoma longifolia* [6]. These compounds have been garnering increasing interest due to numerous activities [7], including antitumor [8,9], antiviral [10], analgesic [11] and anti-inflammatory activities [12]. Although several reports have been published on the insecticidal activities, growth inhibitory activities and/or oviposition deterrence of solvent extracts and β-carboline alkaloids of *P. harmala* against *Tribolium castaneum* [13], *Rhyzopertha dominica* [14], *Plutella xylostella* [15], *Spodoptera littoralis* [16,17], *Schistocerca gregaria* [18] and *Bactrocera oleae* [19], little is known about the bioactivity of harmine against *Spodoptera litura*. In our previous work [20,21], we found that high doses of extract and total alkaloids of *P. harmala* displayed moderate anti-feedant activity against *Spodoptera litura*. Furthermore, we demonstrated that harmine exhibited marginal to weak cytotoxicity against *Spodoptera frugiperda* Sf9 cells [22]. Accordingly, in an attempt to improve toxicity and growth inhibitory activity of β-carboline derivatives against *S. litura*, while, considering that introduction of 1,3,4-oxadiazole would usually potentiate the pharmacological properties of original molecule [23,24,25], we synthesized a series of novel β-carboline 1,3,4-oxadiazole derivatives and evaluated for their in vitro cytotoxicity against Sf9 cells and growth inhibitory activity against *S. litura*.

## 2. Results and Discussion

### 2.1. Chemistry

**Figure 2:**
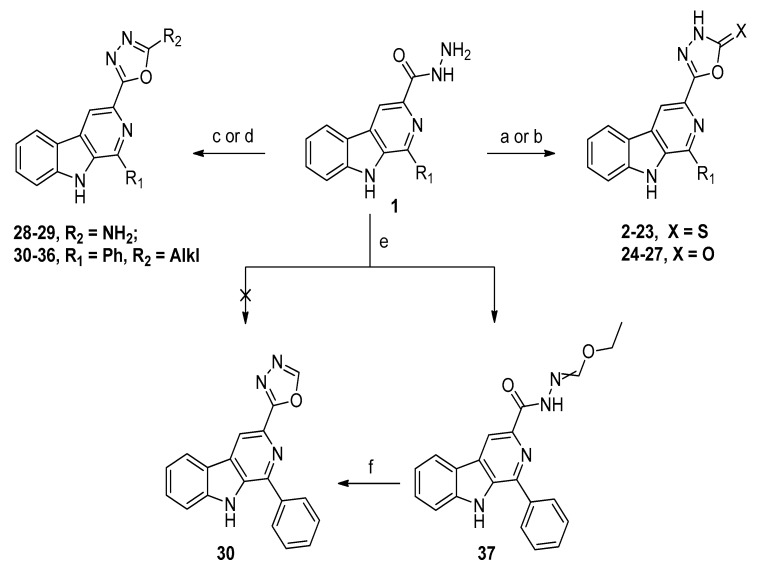
Synthesis of β-carboline 1,3,4-oxadiazole derivatives **2**–**37**. Reagents and conditions: (a) For compounds **2**–**23**: i: CS_2_, KOH, EtOH, reflux, 24 h; ii: acidified 2N HCl; (b) For compounds **24**–**27**: triphosgene, CH_2_Cl_2_, reflux, 2 h; (c) For compounds **28** and **29**: BrCN, NaHCO_3_, dioxane, water, r.t., 4 h; (d) For compounds **30–36**: acid, phosphorus oxychloride, reflux, 4–6 h; (e) triethyl orthoformate, reflux, overnight; and (f) pyridine, reflux, 12 h.

The synthesis of 3-(2-substituted-1,3,4-oxadiazol-5-yl)-β-carbolines are depicted in Figure 2. Briefly, precursor 1-substituted-β-carboline-3-carbohydrazides (**1**) was prepared according to the procedure reported previously [26]. The intermediate **1** could be readily converted into 3-(2-thioxo-1,3,4-oxadiazol-5-yl)-β-carbolines **2**–**23** on treatment with carbon disulfide and potassium hydroxide [27], and into 3-(2-oxo-1,3,4-oxadiazol-5-yl)-β-carbolines **24**–**27** on treatment with triphosgene [28]. Similarly, intermediate 1 furnished desired compounds **28** and **29** by reaction with cyanogen bromide in the presence of sodium bicarbonate at ambient temperature [29]. For preparation of 3-(2-substituted-1,3,4-oxadiazol-5-yl)-β-carbolines **30**–**36**, 1-phenyl-β-carboline-3-carbohydrazide was subjected to reaction with a series of acids, followed by treatment with phosphorus oxychloride under reflux [30]. The compound **30** could be synthesized using other methodology [31]. The 1-phenyl-β-carboline-3-carbohydrazide was reacted with triethyl orthoformate under reflux to give the key intermediate hydrazonoformate **37**, which was used for further cyclization in pyridine under reflux to obtain the desired compound **30**. All newly synthesized compounds were purified by crystallization and their structures were confirmed on the basis of ^1^H NMR, ^13^C NMR and HRMS data, which are given in Section 4. Compounds **3**, **9**, **10**, **13** and **25** were characterized by comparison with NMR data previously reported [27,32]. Compounds **24**–**29** were characterized by the presence of an additional signal at *δ*_H_ 12.69–12.60 or 7.25–7.29, corresponding to the 3′-NH or 2′-NH_2_ on the 1,3,4-oxadiazole scaffold, respectively.

### 2.2. Biological Activity

#### 2.2.1. Anti-Proliferative Activity against Sf9 Cells

All synthesized β-carboline derivatives **2**–**37** were evaluated for their anti-proliferative activities against Sf9 cells and compared with harmine and camptothecin as positive controls. The biological results presented in Table 1 show that all of the target compounds exhibited moderate to potent in vitro cytotoxic activity against Sf9 cells, and some of them were more potent than harmine and camptothecin. Significantly, compounds **5**, **6**, **9**, **11**, **15**, **22** and **37** displayed pronounced anti-proliferative activities. The IC_50_ values of these compounds were 16.25, 12.80, 10.39, 13.83, 14.22, 11.10 and 3.93 μM, respectively, whereas that of camptothecin was 18.95 μM. Intriguingly, the intermediate hydrazonoformate **37** proved to be the most active compound in this study, and the cytotoxic activity was approximately 30-fold more potent than that of the cyclization product **30** and five-fold higher activities than that of camptothecin, which implied further possibilities for lead compound development.

As for the 1,3,4-oxadiazole series **2**–**29**, the effects of different substituent groups at C-1 were investigated. As shown in Table 1, changing the 1-substituent groups could lead to remarkable change in activity. For instance, the activity of compounds with methyl group, such as compounds **2** and **24**, were predominantly lower than those of derivatives with an aromatic or heterocycle group. Moreover, the potency of derivatives with the aromatic group (**3**–**18**) significantly depended upon the nature of substitutes and their position at the aromatic ring. Substitution of electron withdrawing group on the aromatic ring at para position was more potent than that of electron donating group, while compound **9** bearing 4-OCH_3_-Ph group displayed the highest cytotoxic activity (IC_50_ = 10.39 μM). For the effect of substituted position on the phenyl ring, mono-substitution on the aromatic ring at different position (**5**, **6** and **10**–**12**) showed similar potency. By contrast to mono-substituted compounds, 3,4-disubstituted derivatives (**14** and **16–18**) were much less potent, whereas 3,4,5-trisubstituted compound (**15**) showed comparable potency to the mono-substituted compounds. Comparing with phenyl ring substituted compounds, condensed ring substituted compounds (**19**–**23**) displayed equivalent activity.

For the effect of different substituent groups at 2-position on 1,3,4-oxadiazole scaffold (**24**–**36**), the available data analysis suggested that there was no significant difference on cytotoxic activity when changing thioxo group to oxo group (**25**–**27**) or amino group (**28** and **29**) except for compound **24**. However, Compounds **30**–**34** with aliphatic groups or aromatic groups in the R_2_ position showed marginal to weak cytotoxicity (IC_50_ 66.63 to 144.66 μM), even compounds **35** and **36** with 4-CH_3_-Ph and 4-Cl-Ph groups, respectively, were inactive (IC_50_ > 1000 μM), indicating that electronegativity and size of substituents at 2-position on 1,3,4-oxadiazole scaffold are crucial.

#### 2.2.2. Compounds **5** and **37** Could Induce Cell Apoptosis in Sf9 Cells

**Figure 3:**
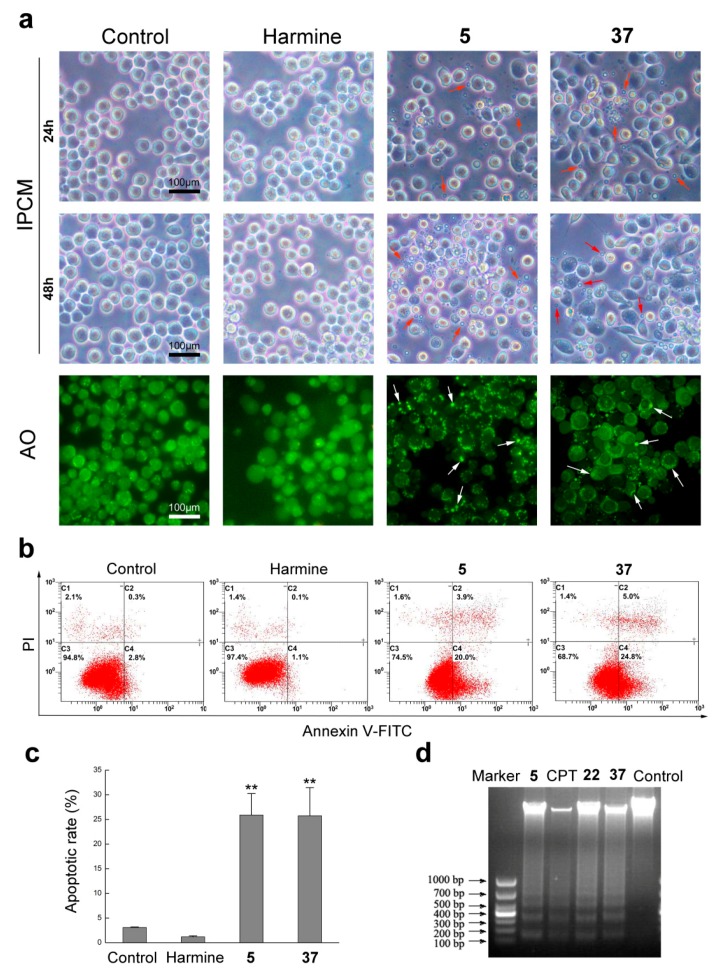
Analysis of apoptosis in Sf9 cells treated with 12.5 μg mL^−1^ **5** and **37**. (**a**) Representative photographs of cell morphological change induced by harmine, **5** and **37** for 24 and 48 h, respectively. IPCM: inverted phase contrast microscopy; AO: acridine orange. Red and white arrows represent apoptosis bodies; (**b**,**c**) Apoptotic rate of cells induced by 12.5 μg mL^−1^ **5** and **37** for 24 h; (**d**) Agarose gel electrophoresis analysis of genomic DNA of cells treated by 12.5 μg mL^−1^ **5**, **22** and **37** with CPT as a positive control. CPT: camptotothecin. Note: (**) *p* < 0.01 in comparison to the control.

We found that compounds **5** and **37** had apoptosis effect on Sf9 cells through morphology observation after treatment with the 36 β-carboline derivatives. We treated Sf9 cells with various concentrations of compound **5** for 24 h and observed cellular morphological changes under inverted phase contrast microscope (IPCM). As shown in Figure S1, addition of compound **5** to culture Sf9 cells was found to induce membrane blebbing and a small amount of apoptotic bodies and floating cells at 10 μg mL^−1^, and apoptotic bodies increased while adherent cells decreased with increasing drug concentrations, whereas no apoptotic bodies were observed in 20 μg mL^−1^-treated group. Apoptosis was also confirmed by staining with acridine orange (AO); the non-apoptotic cells were stained uniformly green and the apoptotic cells had evident orange particles in their nuclei due to nuclear condensation or nuclear DNA fragmentation, which were typical early apoptotic characteristics. Analogously, we treated Sf9 cells with 12.5 μg mL^−1^ harmine, compounds **5** and **37** for 24 and 48 h and found that compounds **5** and **37** could induce apoptotic bodies, and apoptotic bodies increased with the extension of induction time (Figure 3a). However, both harmine-treated and untreated cells did not exhibit typical morphologic features of apoptosis, such as cell shrinkage or formation of apoptotic bodies, until the exposure time was extended to 48 h. Similar results could be obtained by AO staining. Additionally, DNA fragmentation was detected through agarose gel electrophoresis and DNA ladder was observed clearly after 24 h incubation with compounds **5** and **37** (Figure 3d), while no DNA fragmentation were observed after treatment with various concentrations of harmine (Figure S2). Subsequently, we detected the cell apoptosis using annexin V-FITC/PI staining and flow cytometry. As shown in Figure 3b,c, the apoptotic rate of compounds **5** and **37** treatment increased dramatically in comparison with untreated and harmine-treated groups, and the proportion of apoptotic cells was 23.9% and 29.8%, respectively. These results suggested that compounds **5** and **37** could cause apoptosis in Sf9 cells and more effectively than harmine.

#### 2.2.3. Compounds **5** and **37** Could Induce Cell Cycle Arrest in Sf9 Cells

**Figure 4:**
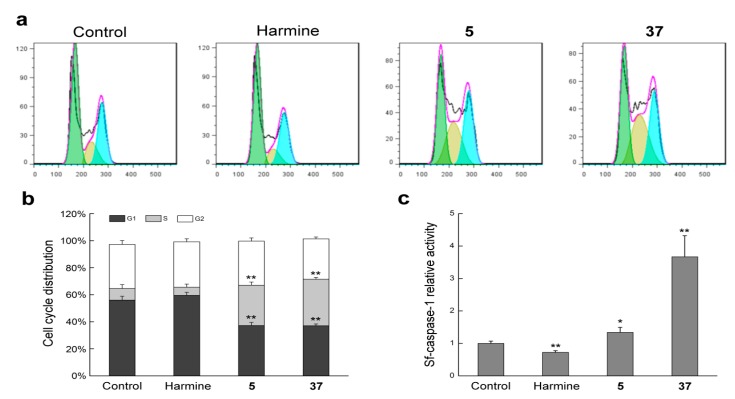
Compounds **5** and **37** induced cell cycle arrest and stimulated Sf-caspase-1 activation in Sf9 cells. (**a**) Cell cycle analysis by flow cytometry. Cells were treated with 12.5 μg mL^−1^ harmine, **5** and **37** for 24 h; (**b**) The percentage of cells in the G1, S and G2 phases of the cell cycle; (**c**) Sf-caspase-1 activity of Sf9 cells after treatment with 12.5 μg mL^−1^ harmine, **5** and **37** for 24 h. Note: (*) *p* < 0.05 and (**) *p* < 0.01 in comparison to the control.

We next assessed the cell cycle distribution of Sf9 cells by flow cytometry. Compounds **5** and **37** treated groups exhibited a high percentage of cells in S phase as compared to the harmine-treated and untreated groups with a concomitant reduction of cell numbers in G_1_ phase, which suggested that compounds **5** and **37** could arrest the cell cycle at the S phase (Figure 4a,b).

#### 2.2.4. Compounds **5** and **37** Could Simulate Sf-Caspase-1 Activation

Caspases play a crucial role in controlling cell death. Executioner caspases (including caspase-3) were activated by initiator caspases (such as caspase-9) and subsequently coordinate their activity to affect main structural proteins and activate other enzymes, resulting in cell apoptosis. Sf-caspase-1, which has the same activity as the mammalian caspase-3, is the main effector caspase in Sf9 cells [33]. To identify whether Sf-caspase-1 was involved in the apoptotic mechanism of compounds **5** and **37** in Sf9 cells, we assessed the Sf-caspase-1 activity. The results demonstrated that compound **5** displayed modest efficacy in Sf-caspase-1 activity; notably, Sf-caspase-1 activity increased significantly and remains almost 3-fold higher in the compound **37** treated cells than that in untreated cells, whereas an obvious decrease in Sf-caspase-1 activity of harmine treated group was observed, suggesting that compounds **5** and **37** could induce Sf-caspase-1 activity in Sf9 cells (Figure 4c).

#### 2.2.5. Compounds **5** and **37** Could Inhibit Larvae Growth of *S. litura*

**Figure 5:**
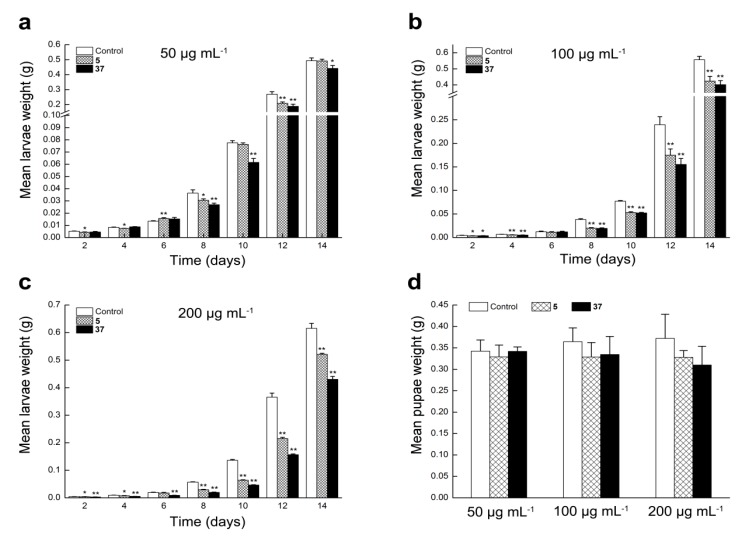
The growth inhibitory activity of compounds **5** and **37** against *S. litura*. (**a**–**c**) Mean larvae weight of *S. Litura* after treatment with **5** and **37** for two weeks at the concentrations of 50, 100 and 200 μg mL^−1^, respectively. (**d**) Mean pupae weight of *S. Litura* after treatment with **5** and **37** at the concentrations of 50, 100 and 200 μg mL^−1^, respectively. Note: (*) *p* < 0.05 and (**) *p* < 0.01 in comparison to the control.

In previous studies, effects of extracts and alkaloid of *P. harmal* on lepidopteran insects were investigated. El-Gengaihi et al. [16] and Shonouda et al. [17] reported that harmine alkaloid caused an increase in larval period and larval mortality but a decrease in the percentage of pupation of *S. littoralis*. Additionally, Abbasipour et al. [15] reported that the extract of *P. harmal* displayed pronounced effect on larval and pupal weight, oviposition deterrence, percent pupation, egg hatching and adult emergence of *P. xylostella*. These findings indicated that β-carboline alkaloids might inhibit growth of lepidopteran larvae. Therefore, based on the anti-feedant activity against *S. litura* in our previous studies, we further evaluated the larvicidal activity and growth inhibitory activity of synthesized β-carboline derivatives against *S. litura*. Unfortunately, there was no significant toxicity of all synthesized compounds against *S. litura* at 500 μg mL^−1^. Subsequently, compounds **5** and **37**, with superior cytotoxicity and apoptosis property, were chosen to evaluate the growth inhibitory activity against 3rd instar *S. litura* and the results is shown in Figure 5. We assessed the effect of compounds **5** and **37** on larvae weight at various concentrations and showed that mean larvae weight of **5**-treated and **37**-treated groups significantly decreased as compared to the control group, and the difference in larvae weight between treated and untreated groups was positively correlated with drug concentrations. Interestingly, moreover, the difference in larvae weight between **5**-treated and **37**-treated groups was consistent with their cytotoxicity against Sf9 cells (Figure 5a–c). In addition, treatment with compounds **5** and **37** at various concentrations led to reduction in the weight of pupae (Figure 5d), but did not significantly affect the percentage of pupation and adult emergence, which was similar to what Jbilou et al. described [13].

## 3. Materials and Methods

### 3.1. Chemistry

All reagents and solvents were of reagent grade or purified according to standard methods before use. Analytical thin-layer chromatography and preparative thin-layer chromatography were performed with silica gel plates using silica gel 60 GF254 (Qingdao Haiyang Chemical Co., Ltd., Qingdao, Shandong Province, China). ^1^H-NMR and ^13^C NMR spectra were recorded at 600 MHz on a Bruker Avance-600 superconducting nuclear magnetic resonance instrument using TMS as a reference (Bruker Company, Gemany). High-resolution mass spectrometry (HRMS) data were obtained on an Uplc1290-6540B Q-TOF LC/MS instrument (Agilent Co., Santa Clara, CA, USA). The key intermediate 1-substituted-β-carboline-3-carbohydrazide derivatives **1** were prepared according to the procedure reported previously [26].

#### 3.1.1. General Synthetic Procedure for Target Compounds **2**–**23**

A mixture of 1-substituted-β-carboline-3-carbohydrazide derivatives **1** (1 mmol), potassium hydroxide (84 mg, 1.5 mmol) and carbon disulfide (0.3 mL, 5 mmol) in absolute ethanol (10 mL) was refluxed with stirring for 24 h. Then, the solvent was removed by evaporation under reduced pressure. The residue obtained was diluted with water and acidified with 2N HCl solution. The product was collected by filtration, washed with water, dried and recrystallized from ethanol.

*1-Methyl-3-(2-thioxo-1,3,4-oxadiazol-5-yl)-9H-pyrido[3,4-b]indole* (**2**). Yield: 78%; ^1^H NMR (600 MHz, DMSO-*d*_6_) δ: 12.09 (s, 1H, 9-NH), 8.71 (s, 1H, 4-H), 8.40 (d, *J* = 7.9 Hz, 1H, 5-H), 7.66 (d, *J* = 8.2 Hz, 1H, 8-H), 7.60 (t, *J* = 8.1 Hz, 1H, 7-H), 7.31 (t, *J* = 7.1 Hz, 1H, 6-H), 2.85 (s, 3H, 1-CH3); ^13^C NMR (151 MHz, DMSO-*d*_6_) δ: 177.90, 161.71, 143.67, 141.26, 136.05, 130.53, 129.08, 127.50, 122.77, 121.52, 120.66, 113.55, 112.78, 20.82; HRMS Calcd for 283.0648 (C_14_H_10_N_4_OS, [M + H]^+^), found 283.0862.

*1-Phenyl-3-(2-thioxo-1,3,4-oxadiazol-5-yl)-9H-pyrido[3,4-b]indole* (**3**). Yield: 72%; ^1^H NMR (600 MHz, DMSO-*d*_6_) δ: 11.99 (s, 1H, 9-NH), 8.88 (s, 1H, 4-H), 8.48 (d, *J* = 7.9 Hz, 1H, 5-H), 8.07–8.04 (m, 2H, Ar-H), 7.71 (d, *J* = 8.2 Hz, 1H, 8-H), 7.67 (t, *J* = 7.6 Hz, 2H, Ar-H), 7.64–7.58 (m, 2H, 7-H, Ar-H), 7.35 (t, *J* = 7.5 Hz, 1H, 6-H); ^13^C NMR (151 MHz, DMSO-*d*_6_) δ: 177.60, 161.14, 142.82, 141.61, 137.18, 134.02, 130.93, 129.53, 129.22, 128.98, 128.90, 128.62, 122.25, 120.88, 120.50, 113.94, 112.82; HRMS Calcd for 345.0805 (C_19_H_12_N_4_OS, [M + H]^+^), found 345.1062.

*1-(4-Methylphenyl)-3-(2-thioxo-1,3,4-oxadiazol-5-yl)-9H-pyrido[3,4-b]indole* (**4**). Yield: 74%; ^1^H NMR (600 MHz, DMSO-*d*_6_) δ: 11.92 (s, 1H, 9-NH), 8.82 (s, 1H, 4-H), 8.44 (d, *J* = 7.4 Hz, 1H, 5-H), 7.95 (d, *J* = 7.3 Hz, 2H, Ar-H), 7.70 (d, *J* = 8.0 Hz, 1H, 8-H), 7.64–7.58 (m, 1H, 7-H), 7.46 (d, *J* = 7.3 Hz, 2H, Ar-H), 7.36–7.30 (m, 1H, 6-H), 2.46 (s, 3H, 4′-CH3); ^13^C NMR (151 MHz, DMSO-*d*_6_) δ: 177.60, 161.15, 142.86, 141.53, 138.74, 134.37, 133.91, 130.89, 129.39, 128.85, 128.46, 122.14, 120.87, 120.40, 113.63, 112.77, 20.94; HRMS Calcd for 359.0961 (C_20_H_14_N_4_OS, [M + H]^+^), found 359.0962.

*1-(4-Fluorophenyl)-3-(2-thioxo-1,3,4-oxadiazol-5-yl)-9H-pyrido[3,4-b]indole* (**5**). Yield: 65%; ^1^H NMR (600 MHz, DMSO-*d*_6_) δ: 11.99 (s, 1H, 9-NH), 8.84 (s, 1H, 4-H), 8.45 (d, *J* = 7.8 Hz, 1H, 5-H), 8.09 (dd, *J* = 8.3, 5.6 Hz, 2H, Ar-H), 7.69 (d, *J* = 8.1 Hz, 1H, 8-H), 7.65–7.60 (m, 1H, 7-H), 7.48 (t, *J* = 8.7 Hz, 2H, Ar-H), 7.34 (t, *J* = 7.4 Hz, 1H, 6-H); ^13^C NMR (151 MHz, DMSO-*d*_6_) δ: 177.62, 161.10, 141.83, 141.64, 133.97, 133.63, 130.93, 130.91, 130.87, 129.65, 129.09, 122.31, 120.90, 120.59, 115.90, 115.76, 113.99, 112.81; HRMS Calcd for 363.0710 (C_19_H_11_FN_4_OS, [M + H]^+^), found 363.0819.

*1-(4-Chlorophenyl)-3-(2-thioxo-1,3,4-oxadiazol-5-yl)-9H-pyrido[3,4-b]indole* (**6**). Yield: 80%; ^1^H NMR (600 MHz, DMSO-*d*_6_) δ: 12.03 (s, 1H, 9-NH), 8.89 (s, 1H, 4-H), 8.48 (d, *J* = 7.9 Hz, 1H, 5-H), 8.08 (d, *J* = 8.5 Hz, 2H, Ar-H), 7.75–7.68 (m, 3H, 8-H, Ar-H), 7.66–7.61 (m, 1H, 7-H), 7.35 (t, *J* = 7.4 Hz, 1H, 6-H). ^13^C NMR (151 MHz, DMSO-*d*_6_) δ: 177.61, 161.02, 141.63, 141.44, 135.92, 133.99, 130.93, 130.43, 129.76, 129.12, 128.91, 122.30, 120.85, 120.60, 114.17, 112.77. HRMS Calcd for 379.0415 (C_19_H_11_ClN_4_OS, [M + H]^+^), found 379.0604.

*1-(4-Bromophenyl)-3-(2-thioxo-1,3,4-oxadiazol-5-yl)-9H-pyrido[3,4-b]indole* (**7**). Yield: 78%; ^1^H NMR (600 MHz, DMSO-*d*_6_) δ: 12.02 (s, 1H, 9-NH), 8.89 (s, 1H, 4-H), 8.48 (d, *J* = 7.8 Hz, 1H, 5-H), 8.01 (d, *J* = 8.3 Hz, 2H, Ar-H), 7.85 (d, *J* = 8.3 Hz, 2H, Ar-H), 7.70 (d, *J* = 8.2 Hz, 1H, 8-H), 7.63 (t, *J* = 7.6 Hz, 1H, 7-H), 7.35 (t, *J* = 7.4 Hz, 1H, 6-H); ^13^C NMR (151 MHz, DMSO-*d*_6_) δ: 177.64, 161.04, 141.65, 141.55, 136.30, 133.99, 131.87, 131.01, 130.71, 129.80, 129.16, 122.73, 122.34, 120.87, 120.64, 114.22, 112.79. HRMS Calcd for 422.9910 (C_19_H_11_BrN_4_OS, [M + H]^+^), found 423.0025.

*1-(4-Trifluoromethylphenyl)-3-(2-thioxo-1,3,4-oxadiazol-5-yl)-9H-pyrido[3,4-b]indole* (**8**). Yield: 76%; ^1^H NMR (600 MHz, DMSO-*d*_6_) δ: 12.08 (s, 1H, 9-NH), 8.92 (s, 1H, 4-H), 8.49 (d, *J* = 7.9 Hz, 1H, 5-H), 8.25 (d, *J* = 8.1 Hz, 2H, Ar-H), 8.01 (d, *J* = 8.2 Hz, 2H, Ar-H), 7.69 (d, *J* = 8.2 Hz, 1H, 8-H), 7.64 (t, *J* = 7.6 Hz, 1H, 7-H), 7.36 (t, *J* = 7.4 Hz, 1H, 6-H); ^13^C NMR (151 MHz, DMSO-*d*_6_) δ: 177.62, 160.92, 141.68, 140.97, 134.16, 131.03, 129.96, 129.48, 129.21, 125.72, 125.70, 122.34, 120.79, 120.65, 114.53, 112.74. HRMS Calcd for 413.0678 (C_20_H_11_F_3_N_4_OS, [M + H]^+^), found 413.0940.

*1-(4-Methoxyphenyl)-3-(2-thioxo-1,3,4-oxadiazol-5-yl)-9H-pyrido[3,4-b]indole* (**9**). Yield: 60%; ^1^H NMR (600 MHz, DMSO-*d*_6_) δ: 11.88 (s, 1H, 9-NH), 8.76 (s, 1H, 4-H), 8.43 (d, *J* = 7.8 Hz, 1H, 5-H), 8.02 (d, *J* = 8.6 Hz, 2H, Ar-H), 7.70 (d, *J* = 8.2 Hz, 1H, 8-H), 7.60 (t, *J* = 7.5 Hz, 1H, 7-H), 7.32 (t, *J* = 7.4 Hz, 1H, 6-H), 7.20 (d, *J* = 8.6 Hz, 2H, Ar-H), 3.89 (s, 3H, 4′-OCH3); ^13^C NMR (151 MHz, DMSO-*d*_6_) δ: 178.03, 161.34, 160.04, 142.51, 141.52, 133.55, 131.56, 130.15, 129.94, 129.74, 129.36, 128.71, 122.07, 120.94, 120.28, 114.24, 114.09, 112.89, 112.73, 55.35; HRMS Calcd for 375.0910 (C_20_H_14_N_4_O_2_S, [M + H]^+^), found 375.1054.

*1-(2-Chlorophenyl)-3-(2-thioxo-1,3,4-oxadiazol-5-yl)-9H-pyrido[3,4-b]indole* (**10**). Yield: 64%; ^1^H NMR (600 MHz, DMSO-*d*_6_) δ: 11.81 (s, 1H, 9-NH), 8.91 (s, 1H, 4-H), 8.47 (d, *J* = 7.9 Hz, 1H, 5-H), 7.72 (d, *J* = 8.0 Hz, 1H, 8-H), 7.67–7.57 (m, 5H, 7-H, Ar-H), 7.35–7.31 (m, 1H, 6-H); ^13^C NMR (151 MHz, DMSO-*d*_6_) δ: 177.83, 161.13, 141.83, 141.49, 136.11, 134.87, 132.48, 131.89, 130.82, 129.80, 129.10, 128.74, 127.56, 122.45, 120.80, 120.42, 114.34, 112.53; HRMS Calcd for 379.0415 (C_19_H_11_ClN_4_OS, [M + H]^+^), found 379.0415.

*1-(3-Chlorophenyl)-3-(2-thioxo-1,3,4-oxadiazol-5-yl)-9H-pyrido[3,4-b]indole* (**11**). Yield: 73%; ^1^H NMR (600 MHz, DMSO-*d*_6_) δ: 12.06 (s, 1H, 9-NH), 8.87 (s, 1H, 4-H), 8.47 (d, *J* = 7.9 Hz, 1H, 5-H), 8.03 (s, 1H, Ar-H), 8.01 (d, *J* = 7.4 Hz, 1H, 8-H), 7.66 (m, 4H, 7-H, Ar-H), 7.35 (t, *J* = 7.4 Hz, 1H, 6-H); ^13^C NMR (151 MHz, DMSO-*d*_6_) δ: 177.59, 160.95, 141.66, 141.03, 139.14, 134.00, 133.62, 130.93, 130.76, 129.88, 129.15, 129.09, 128.25, 127.30, 122.32, 120.82, 120.60, 114.37, 112.77; HRMS Calcd for 379.0415 (C_19_H_11_ClN_4_OS, [M + H]^+^), found 379.0415.

*1-(3-Fluorophenyl)-3-(2-thioxo-1,3,4-oxadiazol-5-yl)-9H-pyrido[3,4-b]indole* (**12**). Yield: 54%; ^1^H NMR (600 MHz, DMSO-*d*_6_) δ: 12.03 (s, 1H, 9-NH), 8.87 (s, 1H, 4-H), 8.47 (d, *J* = 7.9 Hz, 1H, 5-H), 7.89 (d, *J* = 7.7 Hz, 1H, Ar-H), 7.82 (d, *J* = 9.9 Hz, 1H, 8-H), 7.72 – 7.67 (m, 2H, Ar-H), 7.63 (t, *J* = 7.6 Hz, 1H, 7-H), 7.43 (dd, *J* = 8.6, 2.5 Hz, 1H, Ar-H), 7.35 (t, *J* = 7.5 Hz, 1H, 6-H); ^13^C NMR (151 MHz, DMSO-*d*_6_) δ: 177.60, 163.16, 161.54, 141.65, 141.17, 139.43 (139.38), 133.96, 130.97, 130.92, 129.87, 129.14, 124.78, 122.29, 120.82, 120.60, 116.12, 115.98, 115.38, 115.24, 114.31, 112.79; HRMS Calcd for 363.0710 (C_19_H_11_FN_4_OS, [M + H]^+^), found 363.0712.

*1-(3-Nitrophenyl)-3-(2-thioxo-1,3,4-oxadiazol-5-yl)-9H-pyrido[3,4-b]indole* (**13**). Yield: 64%; ^1^H NMR (600 MHz, DMSO-*d*_6_) δ: 12.12 (s, 1H, 9-NH), 8.88 (s, 1H, 4-H), 8.79 (s, 1H, Ar-H), 8.47 (d, *J* = 6.5 Hz, 2H, Ar-H), 8.41 (d, *J* = 7.7 Hz, 1H, 5-H), 7.94 (t, *J* = 7.4 Hz, 1H, Ar-H), 7.68 (d, *J* = 7.6 Hz, 1H, 8-H), 7.66–7.61 (m, 1H, 7-H), 7.35 (t, *J* = 6.8 Hz, 1H, 6-H); ^13^C NMR (151 MHz, DMSO-*d*_6_) δ: 178.00, 160.97, 148.18, 141.68, 139.98, 138.65, 135.00, 133.97, 131.68, 130.50, 130.19, 129.25, 123.69, 123.31, 122.39, 120.84, 120.62, 114.35, 112.65; HRMS Calcd for 390.0655 (C_19_H_11_N_5_O_3_S, [M + H]^+^), found 390.0657.

*1-(3,4-Dimethoxyphenyl)-3-(2-thioxo-1,3,4-oxadiazol-5-yl)-9H-pyrido[3,4-b]indole* (**14**). Yield: 59%; ^1^H NMR (600 MHz, DMSO-*d*_6_) δ: 11.95 (s, 1H, 9-NH), 8.82 (s, 1H, 4-H), 8.45 (d, *J* = 7.9 Hz, 1H, 5-H), 7.70 (d, *J* = 8.2 Hz, 1H, 8-H), 7.61–7.59 (m, 2H, Ar-H), 7.57 (d, *J* = 1.9 Hz, 1H, 7-H), 7.35–7.32 (m, 1H, 6-H), 7.23 (d, *J* = 8.4 Hz, 1H, Ar-H), 3.90 (s, 3H, 3′-OCH3), 3.89 (s, 3H, 4′-OCH3); ^13^C NMR (151 MHz, DMSO-*d*_6_) δ: 177.56, 161.21, 149.84, 148.90, 143.13, 141.52, 133.97, 130.78, 129.78, 129.24, 128.86, 122.22, 121.36, 120.97, 120.46, 113.57, 112.83, 112.02 (112.98), 55.76, 55.59; HRMS Calcd for 405.1016 (C_21_H_16_N_4_O_3_S, [M + H]^+^), found 405.1013.

*1-(3,4,5-Trimethoxyphenyl)-3-(2-thioxo-1,3,4-oxadiazol-5-yl)-9H-pyrido[3,4-b]indole* (**15**). Yield: 81%; ^1^H NMR (600 MHz, DMSO-*d*_6_) δ: 11.85 (s, 1H, 9-NH), 8.78 (s, 1H, 4-H), 8.44 (d, *J* = 7.8 Hz, 1H, 5-H), 7.67 (d, *J* = 8.2 Hz, 1H, 8-H), 7.62–7.58 (m, 1H, 7-H), 7.32 (dd, *J* = 11.4, 4.4 Hz, 1H, 6-H), 7.22 (s, 2H, Ar-H), 3.93 (s, 6H, 3′, 5′-OCH3), 3.79 (s, 3H, 4′-OCH3); ^13^C NMR (151 MHz, DMSO-*d*_6_) δ: 178.99, 161.60, 153.08, 142.68, 141.49, 138.21, 133.41, 133.04, 129.40, 128.60, 122.03, 120.98, 120.06, 112.61, 112.43, 106.03, 60.06, 55.97; HRMS Calcd for 435.1122 (C_22_H_18_N_4_O_4_S, [M + H]^+^), found 435.1258.

*1-(3,4-Difluorophenyl)-3-(2-thioxo-1,3,4-oxadiazol-5-yl)-9H-pyrido[3,4-b]indole* (**16**). Yield: 76%; ^1^H NMR (600 MHz, DMSO-*d*_6_) δ: 12.04 (s, 1H, 9-NH), 8.85 (s, 1H, 4-H), 8.46 (d, *J* = 7.9 Hz, 1H, 5-H), 8.06–8.02 (m, 1H, Ar-H), 7.89 (dd, *J* = 8.2, 3.7 Hz, 1H, Ar-H), 7.73–7.67 (m, 2H, 8-H, Ar-H), 7.65–7.61 (m, 1H, 7-H), 7.34 (t, *J* = 7.0 Hz, 1H, 6-H); ^13^C NMR (151 MHz, DMSO-*d*_6_) δ: 177.63, 160.94, 141.67, 133.91, 130.89, 129.92, 129.23, 125.85, 122.35, 120.83, 120.68, 118.11, 118.00, 117.84, 117.72, 114.34, 112.77; HRMS Calcd for 381.0616 (C_19_H_10_F_2_N_4_OS, [M + H]^+^), found 381.0621.

*1-(3,4-Dichlorophenyl)-3-(2-thioxo-1,3,4-oxadiazol-5-yl)-9H-pyrido[3,4-b]indole* (**17**). Yield: 75%; ^1^H NMR (600 MHz, DMSO-*d*_6_) δ: 12.08 (s, 1H, 9-NH), 8.85 (s, 1H, 4-H), 8.46 (d, *J* = 7.9 Hz, 1H, 5-H), 8.20 (d, *J* = 2.0 Hz, 1H, Ar-H), 8.01 (dd, *J* = 8.3, 2.0 Hz, 1H, Ar-H), 7.89 (d, *J* = 8.3 Hz, 1H, Ar-H), 7.69 (d, *J* = 8.2 Hz, 1H, 8-H), 7.63 (t, *J* = 7.6 Hz, 1H, 7-H), 7.34 (t, *J* = 7.4 Hz, 1H, 6-H); ^13^C NMR (151 MHz, DMSO-*d*_6_) δ: 177.59, 160.84, 141.64, 139.93, 137.56, 133.95, 131.89, 131.64, 131.00, 130.94, 130.30, 130.00, 129.21, 128.81, 122.33, 120.78, 120.64, 114.49, 112.71; HRMS Calcd for 413.0025 (C_19_H_10_Cl_2_N_4_OS, [M + H]^+^), found 413.0029.

*1-(3-Fluoro-4-methoxyphenyl)-3-(2-thioxo-1,3,4-oxadiazol-5-yl)-9H-pyrido[3,4-b]indole* (**18**). Yield: 69%; ^1^H NMR (600 MHz, DMSO-*d*_6_) δ: 11.95 (s, 1H, 9-NH), 8.80 (s, 1H, 4-H), 8.44 (d, *J* = 7.9 Hz, 1H, 5-H), 7.89 (s, 1H, Ar-H), 7.87 (d, *J* = 3.2 Hz, 1H, Ar-H), 7.70 (d, *J* = 8.2 Hz, 1H, 8-H), 7.62 (t, *J* = 7.6 Hz, 1H, 7-H), 7.43 (t, *J* = 8.7 Hz, 1H, Ar-H), 7.33 (t, *J* = 7.4 Hz, 1H, 6-H), 3.97 (s, 3H, 4′-OCH3); ^13^C NMR (151 MHz, DMSO-*d*_6_) δ: 178.08, 161.23, 152.30, 150.68, 148.03 (147.96), 141.60, 141.14, 133.55, 129.71, 128.93, 125.21, 122.20, 120.92, 120.44, 116.01, 115.88, 114.04, 112.75, 56.27; HRMS Calcd for 393.0816 (C_20_H_13_FN_4_O_2_S, [M + H]^+^), found 393.0814.

*1-(1,3-Benzodioxole-5-yl)-3-(2-thioxo-1,3,4-oxadiazol-5-yl)-9H-pyrido[3,4-b]indole* (**19**). Yield: 80%; ^1^H NMR (600 MHz, DMSO-*d*_6_) δ: 11.92 (s, 1H, 9-NH), 8.80 (s, 1H, 4-H), 8.44 (d, *J* = 7.9 Hz, 1H, 5-H), 7.70 (d, *J* = 8.2 Hz, 1H, 8-H), 7.62–7.56 (m, 3H, 7-H, Ar-H), 7.33 (t, *J* = 7.5 Hz, 1H, 6-H), 7.19 (d, *J* = 7.9 Hz, 1H, Ar-H), 6.18 (s, 2H, -OCH2O-); ^13^C NMR (151 MHz, DMSO-*d*_6_) δ: 177.73, 161.18, 148.13, 147.68, 142.33, 141.51, 133.64, 131.22, 131.04, 129.45, 128.84, 122.85, 122.15, 120.90, 120.39, 113.39, 112.77, 108.72, 108.65, 101.47; HRMS Calcd for 389.0703 (C_20_H_12_N_4_O_3_S, [M + H]^+^), found 389.0709.

*1-(2,3-Dihydro-benzofuran-5-yl)-3-(2-thioxo-1,3,4-oxadiazol-5-yl)-9H-pyrido[3,4-b]indole* (**20**). Yield: 70%; ^1^H NMR (600 MHz, DMSO-*d*_6_) δ: 11.87 (s, 1H, 9-NH), 8.76 (s, 1H, 4-H), 8.43 (d, *J* = 7.9 Hz, 1H, 5-H), 7.93 (s, 1H, Ar-H), 7.82 (d, *J* = 8.2 Hz, 1H, Ar-H), 7.70 (d, *J* = 8.2 Hz, 1H, 8-H), 7.62–7.58 (m, 1H, 7-H), 7.32 (t, *J* = 7.4 Hz, 1H, 6-H), 7.01 (d, *J* = 8.2 Hz, 1H, Ar-H), 4.66 (t, *J* = 8.7 Hz, 2H, -OCH2CH2-), 3.35 (t, *J* = 8.6 Hz, 2H, -OCH2CH2-); ^13^C NMR (151 MHz, DMSO-*d*_6_) δ: 177.84, 161.31, 160.68, 143.00, 141.46, 133.58, 131.24, 129.65, 129.27, 128.72, 128.12, 125.53, 122.10, 120.96, 120.30, 112.93, 112.71, 109.08, 71.49, 29.06; HRMS Calcd for 387.0910 (C_21_H_14_N_4_O_2_S, [M + H]^+^), found 387.0914.

*1-(2,3-Dihydro-1,4-benzodioxine-6-yl)-3-(2-thioxo-1,3,4-oxadiazol-5-yl)-9H-pyrido[3,4-b]indole* (**21**). Yield: 68%; ^1^H NMR (600 MHz, DMSO-*d*_6_) δ: 11.92 (s, 1H, 9-NH), 8.79 (s, 1H, 4-H), 8.44 (d, *J* = 7.9 Hz, 1H, 5-H), 7.71 (s, 1H, 8-H), 7.61 (t, *J* = 7.7 Hz, 1H, 7-H), 7.57–7.54 (m, 2H, Ar-H), 7.32 (t, *J* = 7.5 Hz, 1H, 6-H), 7.12 (d, *J* = 8.7 Hz, 1H, Ar-H), 4.36 (s, 4H, -OCH2CH2O-); ^13^C NMR (151 MHz, DMSO-*d*_6_) δ: 177.85, 161.24, 144.44, 143.47, 142.17, 141.54, 133.56, 131.21, 130.46, 129.43, 128.78, 122.09, 121.72, 120.91, 120.34, 117.41, 117.16, 113.19, 112.80, 64.36, 64.16; HRMS Calcd for 403.0859 (C_21_H_14_N_4_O_3_S, [M + H]^+^), found 403.0862.

*1-(2-Naphthyl)-3-(2-thioxo-1,3,4-oxadiazol-5-yl)-9H-pyrido[3,4-b]indole* (**22**). Yield: 78%; ^1^H NMR (600 MHz, DMSO-*d*_6_) δ: 12.09 (s, 1H, 9-NH), 8.87 (s, 1H, 4-H), 8.60 (s, 1H, Ar-H), 8.48 (d, *J* = 7.9 Hz, 1H, 5-H), 8.18 (s, 2H, Ar-H), 8.17–8.13 (m, 1H, Ar-H), 8.08–8.04 (m, 1H, Ar-H), 7.72 (d, *J* = 8.2 Hz, 1H, 8-H), 7.65–7.62 (m, 3H, 7-H, Ar-H), 7.35 (t, *J* = 7.5 Hz, 1H, 6-H); ^13^C NMR (151 MHz, DMSO-*d*_6_) δ: 177.86, 161.23, 142.64, 141.63, 134.57, 134.19, 133.14, 132.80, 131.45, 129.62, 128.96, 128.83, 128.42, 128.00, 127.65, 127.00, 126.51, 126.30, 122.28, 120.96, 120.46, 113.74, 112.75. HRMS Calcd for 395.0961 (C_23_H_14_N_4_OS, [M + H]^+^), found 395.0962.

*1-(6-Quinolinyl)-3-(2-thioxo-1,3,4-oxadiazol-5-yl)-9H-pyrido[3,4-b]indole* (**23**). Yield: 74%; ^1^H NMR (600 MHz, DMSO-*d*_6_) δ: 12.16 (s, 1H, 9-NH), 9.03 (dd, *J* = 4.2, 1.7 Hz, 1H, Ar-H), 8.92 (s, 1H, 4-H), 8.67 (d, *J* = 1.9 Hz, 1H, Ar-H), 8.61 (d, *J* = 8.1 Hz, 1H, Ar-H), 8.50 (d, *J* = 7.9 Hz, 1H, 5-H), 8.43 (dd, *J* = 8.7, 2.0 Hz, 1H, Ar-H), 8.27 (d, *J* = 8.7 Hz, 1H, Ar-H), 7.72 (d, *J* = 8.2 Hz, 1H, 8-H), 7.68 (dd, *J* = 8.2, 4.2 Hz, 1H, Ar-H), 7.65 (t, *J* = 7.1 Hz, 1H, 7-H), 7.37 (t, *J* = 7.9 Hz, 1H, 6-H); ^13^C NMR (151 MHz, DMSO-*d*_6_) δ: 177.60, 161.03, 151.10, 141.87, 141.64, 137.62, 135.11, 134.34, 131.09, 130.16, 129.79, 129.14, 129.03, 128.32, 127.81, 122.37, 122.02, 120.90, 120.61, 114.25, 112.73; HRMS Calcd for 396.0914 (C_22_H_13_N_5_OS, [M + H]^+^), found 396.0916.

#### 3.1.2. General Synthetic Procedure for Target Compounds **24**–**27**

To the solution of corresponding hydrazine derivatives **1** (1 mmol) in dry dichloromethane (10 mL) was added dropwise triphosgene (103.86 mg, 0.35 mmol) in dry dichloromethane (2 mL) under an ice bath and nitrogen atmosphere. After refluxed for 2 h, the solvent was removed by evaporation under reduced pressure, and the product was recrystallized from ethanol.

*1-Methyl-3-(2-oxo-1,3,4-oxadiazol-5-yl)-9H-pyrido[3,4-b]indole* (**24**). Yield: 78%; ^1^H NMR (600 MHz, DMSO-*d*_6_) δ: 12.69 (s, 1H, NH), 12.30 (s, 1H, 9-NH), 8.67 (s, 1H, 4-H), 8.41 (d, *J* = 7.6 Hz, 1H, 5-H), 7.68 (d, *J* = 8.2 Hz, 1H, 8-H), 7.63 (t, *J* = 7.6 Hz, 1H, 7-H), 7.33 (t, *J* = 7.4 Hz, 1H, 6-H), 2.88 (s, 3H, 1-CH3); ^13^C NMR (151 MHz, DMSO-*d*_6_) δ: 163.50, 154.37, 142.54, 141.78, 136.13, 134.98, 129.29, 127.57, 122.46, 121.01, 120.48, 113.88, 112.45, 19.51. HRMS Calcd for 267.0877 (C_14_H_10_N_4_O_2_, [M + H]^+^), found 267.0884.

*1-Phenyl-3-(2-oxo-1,3,4-oxadiazol-5-yl)-9H-pyrido[3,4-b]indole* (**25**). Yield: 87%; ^1^H NMR (600 MHz, DMSO-*d*_6_) δ: 12.60 (s, 1H, NH), 11.91 (s, 1H, 9-NH), 8.75 (s, 1H, 4-H), 8.44 (d, *J* = 7.8 Hz, 1H, 5-H), 8.06–8.03 (m, 2H, Ar-H), 7.69 (d, *J* = 8.1 Hz, 1H, 8-H), 7.66 (t, *J* = 7.6 Hz, 2H, Ar-H), 7.63–7.57 (m, 2H, 7-H, Ar-H), 7.33 (t, *J* = 7.5 Hz, 1H, 6-H); ^13^C NMR (151 MHz, DMSO-*d*_6_) δ: 154.85, 154.54, 142.49, 141.60, 137.36, 133.73, 132.35, 129.57, 129.11, 128.85, 128.59, 122.14, 120.87, 120.32, 112.75; HRMS Calcd for 329.1033 (C_19_H_12_N_4_O_2_, [M + H]^+^), found 329.1310.

*1-(4-Trifluoromethylphenyl)-3-(2-oxo-1,3,4-oxadiazol-5-yl)-9H-pyrido[3,4-b]indole* (**26**). Yield: 83%; ^1^H NMR (600 MHz, DMSO-*d*_6_) δ: 12.65 (s, 1H, NH), 12.03 (s, 1H, 9-NH), 8.81 (s, 1H, 4-H), 8.47 (d, *J* = 7.9 Hz, 1H, 5-H), 8.26 (d, *J* = 8.0 Hz, 2H, Ar-H), 8.01 (d, *J* = 8.1 Hz, 2H, Ar-H), 7.69 (d, *J* = 8.2 Hz, 1H, 8-H), 7.63 (t, *J* = 7.6 Hz, 1H, 7-H), 7.35 (t, *J* = 7.0 Hz, 1H, 6-H); ^13^C NMR (151 MHz, DMSO-*d*_6_) δ: 154.76, 154.29, 141.70, 141.13, 140.62, 133.88, 132.45, 130.02, 129.77, 129.42, 129.06, 125.62, 120.76, 120.44, 113.35, 112.70; HRMS Calcd for 397.0907 (C_20_H_11_F_3_N_4_O_2_, [M + H]^+^), found 397.0910.

*1-(3,4,5-Trimethoxyphenyl)-3-(2-oxo-1,3,4-oxadiazol-5-yl)-9H-pyrido[3,4-b] indole* (**27**). Yield: 90%; ^1^H NMR (600 MHz, DMSO-*d*_6_) δ: 12.62 (s, 1H, NH), 11.90 (s, 1H, 9-NH), 8.71 (s, 1H, 4-H), 8.42 (d, *J* = 7.9 Hz, 1H, 5-H), 7.69 (d, *J* = 8.2 Hz, 1H, 8-H), 7.60 (t, *J* = 7.6 Hz, 1H, 7-H), 7.32 (t, *J* = 7.5 Hz, 1H, 6-H), 7.22 (s, 2H, Ar-H), 3.92 (s, 6H, 3′, 5′-OCH3), 3.79 (s, 3H, 4′-OCH3); ^13^C NMR (151 MHz, DMSO-*d*_6_) δ: 154.77, 154.44, 153.09, 142.84, 141.51, 138.33, 133.75, 132.70, 132.10, 129.30, 128.76, 122.09, 120.88, 120.25, 112.72 (112.67), 106.04, 60.07, 55.97; HRMS Calcd for 419.1350 (C_22_H_18_N_4_O_5_, [M + H]^+^), found 419.1403.

#### 3.1.3. General Synthetic Procedure for Target Compounds **28**–**29**

To a solution of corresponding hydrazine derivatives **1** (1 mmol) in dioxane (5 mL) was added sodium bicarbonate (84 mg, 1 mmol) in water (1.5 mL), and the mixture was stirred at ambient temperature for 10 min, and then cyanogen bromide (81 μL, 1.1 mmol) was added. After stirring for further 4 h, the reaction mixture was diluted with 15 mL water, and the product was collected by filtration, washed with water, dried and crystallized from methanol.

*1-Phenyl-3-(2-amino-1,3,4-oxadiazol-5-yl)-9H-pyrido[3,4-b]indole* (**28**). Yield: 84%; ^1^H NMR (600 MHz, DMSO-*d*_6_) δ: 11.79 (s, 1H, 9-NH), 8.77 (s, 1H, 4-H), 8.40 (d, *J* = 7.9 Hz, 1H, 5-H), 8.04 (d, *J* = 7.2 Hz, 2H, Ar-H), 7.68 (d, *J* = 8.2 Hz, 1H, 8-H), 7.65 (t, *J* = 7.6 Hz, 2H, Ar-H), 7.61–7.56 (m, 2H, 7-H, Ar-H), 7.32 (t, *J* = 7.5 Hz, 1H, 6-H), 7.25 (s, 2H, 2′-NH2); ^13^C NMR (151 MHz, DMSO-*d*_6_) δ: 164.07, 158.41, 142.08, 141.62, 137.50, 133.34, 133.24, 129.76, 128.90, 128.69, 128.53, 121.98, 120.81, 120.08, 112.61, 112.04; HRMS Calcd for 328.1193 (C_19_H_13_N_5_O, [M + H]^+^), found 328.1205.

*1-(4-Trifluoromethylphenyl)-3-(2-amino-1,3,4-oxadiazol-5-yl)-9H-pyrido[3,4-b]indole* (**29**). Yield: 78%; ^1^H NMR (600 MHz, DMSO-*d*_6_) δ: 11.90 (s, 1H, 9-NH), 8.83 (s, 1H, 4-H), 8.42 (d, *J* = 7.8 Hz, 1H, 5-H), 8.23 (s, 2H, Ar-H), 8.00 (d, *J* = 8.1 Hz, 2H, Ar-H), 7.67 (d, *J* = 8.0 Hz, 1H, 8-H), 7.62 (t, *J* = 7.4 Hz, 1H, 7-H), 7.33 (t, *J* = 7.5 Hz, 1H, 6-H), 7.29 (s, 2H, 2′-NH2); ^13^C NMR (151 MHz, DMSO-*d*_6_) δ: 164.15, 158.26, 141.74, 141.38, 140.36, 133.60, 133.43, 130.23, 129.40, 129.02, 125.60 (125.58), 122.16, 120.75, 120.28, 112.82, 112.57; HRMS Calcd for 396.1067 (C_20_H_12_F_3_N_5_O, [M + H]^+^), found 396.1067.

#### 3.1.4. General Synthetic Procedure for Target Compounds **30**–**36**

An equimolar mixture of 1-phenyl-β-carboline-3-carbohydrazide **1** (302 mg, 1 mmol) and various substituted carboxylic acids (1 mmol) in phosphoryl chloride (5 mL) was refluxed for 4–6 h. The reaction mixture was then cooled, poured into crushed ice and neutralized with 5% NaOH solution. The product was collected by filtration, washed with water, dried and recrystallized from methanol.

*1-Phenyl-3-(1,3,4-oxadiazol-5-yl)-9H-pyrido[3,4-b]indole* (**30**). Yield: 76%; ^1^H NMR (600 MHz, DMSO-*d*_6_) δ: 11.97 (s, 1H, 9-NH), 9.39 (s, 1H, 2′-H), 9.03 (s, 1H, 4-H), 8.48 (d, *J* = 7.9 Hz, 1H, 5-H), 8.09–8.07 (m, 2H, Ar-H), 7.71 (d, *J* = 8.2 Hz, 1H, 8-H), 7.67 (t, *J* = 7.6 Hz, 2H, Ar-H), 7.62 (m, 2H, 7-H, Ar-H), 7.35 (t, *J* = 7.1 Hz, 1H, 6-H); ^13^C NMR (151 MHz, DMSO-*d*_6_) δ: 164.44, 154.62, 142.74, 141.68, 137.30, 134.02, 131.94, 129.71, 129.19, 128.96, 128.89, 128.77, 128.65, 122.20, 120.90, 120.45, 114.39, 112.85; HRMS Calcd for 313.1084 (C_19_H_12_N_4_O, [M + H]^+^), found 313.1087.

*1-Phenyl-3-(2-methyl-1,3,4-oxadiazol-5-yl)-9H-pyrido[3,4-b]indole* (**31**). Yield: 75%; ^1^H NMR (600 MHz, DMSO-*d*_6_) δ: 11.93 (s, 1H, 9-NH), 8.97 (s, 1H, 4-H), 8.47 (d, *J* = 7.8 Hz, 1H, 5-H), 8.07–8.05 (m, 2H, Ar-H), 7.70 (d, *J* = 8.2 Hz, 1H, 8-H), 7.67 (t, *J* = 7.6 Hz, 2H, Ar-H), 7.64–7.58 (m, 2H, 7-H, Ar-H), 7.34 (t, *J* = 7.2 Hz, 1H, 6-H), 2.64 (s, 3H, 2′-CH3); ^13^C NMR (151 MHz, DMSO-*d*_6_) δ: 164.75, 163.99, 142.63, 141.66, 137.32, 133.89, 132.24, 129.69, 129.13, 128.91, 128.86, 128.77, 128.64, 122.16, 120.89, 120.39, 113.85, 112.79, 10.78; HRMS Calcd for 327.1240 (C_20_H_14_N_4_O, [M + H]^+^), found 327.1285.

*1-Phenyl-3-(2-ethyl-1,3,4-oxadiazol-5-yl)-9H-pyrido[3,4-b]indole* (**32**). Yield: 80%; ^1^H NMR (600 MHz, DMSO-*d*_6_) δ: 11.91 (s, 1H, 9-NH), 8.96 (s, 1H, 4-H), 8.45 (d, *J* = 7.9 Hz, 1H, 5-H), 8.06 (d, *J* = 7.4 Hz, 2H, Ar-H), 7.70 (d, *J* = 8.2 Hz, 1H, 8-H), 7.66 (t, *J* = 7.6 Hz, 2H, Ar-H), 7.61 (m, 2H, 7-H, Ar-H), 7.33 (t, *J* = 7.4 Hz, 1H, 6-H), 3.00 (q, *J* = 7.5 Hz, 2H, 2′-CH2CH3), 2.50 (s, 3H, 2′-CH2CH3); ^13^C NMR (151 MHz, DMSO-*d*_6_) δ: 167.82, 164.65, 142.64, 141.63, 137.35, 133.89, 132.29, 129.65, 129.09, 128.88, 128.85, 128.74, 128.62, 122.17, 120.88, 120.35, 113.92, 112.76, 18.52, 10.56; HRMS Calcd for 341.1397 (C_21_H_16_N_4_O, [M + H]^+^), found 341.1400.

*1-Phenyl-3-(2-cyclopropyl-1,3,4-oxadiazol-5-yl)-9H-pyrido[3,4-b]indole* (**33**). Yield: 81%; ^1^H NMR (600 MHz, DMSO-*d*_6_) δ: 11.91 (s, 1H, 9-NH), 8.93 (s, 1H, 4-H), 8.44 (d, *J* = 7.8 Hz, 1H, 5-H), 8.06 (d, *J* = 7.3 Hz, 2H, Ar-H), 7.70 (d, *J* = 8.1 Hz, 1H, 8-H), 7.66 (t, *J* = 7.6 Hz, 2H, Ar-H), 7.63–7.57 (m, 2H, 7-H, Ar-H), 7.33 (t, *J* = 7.3 Hz, 1H, 6-H), 2.36 (m, 1H), 1.22–1.12 (m, 4H); ^13^C NMR (151 MHz, DMSO-*d*_6_) δ: 153.38, 149.74, 130.71, 129.84, 126.07, 122.96, 121.55, 119.24, 118.73, 118.53, 118.42, 118.33, 112.59, 111.49, 110.99, 105.27, 104.30, 11.56, 9.76; HRMS Calcd for 353.1397 (C_22_H_16_N_4_O, [M + H]^+^), found 353.1420.

*1-Phenyl-3-(2-phenyl-1,3,4-oxadiazol-5-yl)-9H-pyrido[3,4-b]indole* (**34**). Yield: 91%; ^1^H NMR (600 MHz, DMSO-*d*_6_) δ: 11.96 (s, 1H, 9-NH), 9.08 (s, 1H, 4-H), 8.47 (d, *J* = 7.8 Hz, 1H, 5-H), 8.18–8.15 (m, 2H, Ar-H), 8.11 (d, *J* = 7.3 Hz, 2H, Ar-H), 7.73–7.58 (m, 8H, 8-H, 7-H, Ar-H), 7.35 (t, *J* = 7.3 Hz, 1H, 6-H); ^13^C NMR (151 MHz, DMSO-*d*_6_) δ: 164.78, 164.07, 142.80, 141.63, 137.36, 134.01, 131.97 (131.94), 129.64, 129.46, 129.14, 128.89, 128.67, 126.65, 123.56, 122.15, 120.93, 120.43, 114.47, 112.82; HRMS Calcd for 389.1397 (C_25_H_16_N_4_O, [M + H]^+^), found 389.1895.

*1-Phenyl-3-[2-(4-methylphenyl)-1,3,4-oxadiazol-5-yl]-9H-pyrido[3,4-b]indole* (**35**). Yield: 89%; ^1^H NMR (600 MHz, DMSO-*d*_6_) δ: 11.95 (s, 1H, 9-NH), 9.07 (s, 1H, 4-H), 8.48 (d, *J* = 8.0 Hz, 1H, 5-H), 8.11 (d, *J* = 7.4 Hz, 2H, Ar-H), 8.05 (d, *J* = 7.9 Hz, 2H, Ar-H), 7.72 (d, *J* = 8.1 Hz, 1H, 8-H), 7.68 (t, *J* = 7.5 Hz, 2H, Ar-H), 7.65–7.59 (m, 3H, 7-H, Ar-H), 7.45 (d, *J* = 7.8 Hz, 2H, Ar-H), 7.36 (t, *J* = 7.4 Hz, 1H, 6-H), 2.42 (s, 3H, 4′′-CH3); ^13^C NMR (151 MHz, DMSO-*d*_6_) δ: 164.50, 164.11, 142.75, 142.03, 141.60, 137.34, 133.94, 132.01, 129.93, 129.61, 129.06, 128.83, 128.61, 126.55, 122.08, 120.89, 120.77, 120.35, 114.32, 112.77, 21.11; HRMS Calcd for 403.1553 (C_26_H_18_N_4_O, [M + H]^+^), found 403.1555.

*1-Phenyl-3-[2-(4-chlorophenyl)-1,3,4-oxadiazol-5-yl]-9H-pyrido[3,4-b]indole* (**36**). Yield: 84%; ^1^H NMR (600 MHz, DMSO-*d*_6_) δ: 11.99 (s, 1H, 9-NH), 9.12 (s, 1H, 4-H), 8.49 (d, *J* = 7.9 Hz, 1H, 5-H), 8.19 (d, *J* = 8.6 Hz, 2H, Ar-H), 8.11 (d, *J* = 7.0 Hz, 2H, Ar-H) , 7.74 (d, *J* = 8.7 Hz, 2H, Ar-H), 7.70 (dd, *J* = 15.3, 7.9 Hz, 3H, 8-H, Ar-H), 7.66–7.59 (m, 2H, 7-H, Ar-H), 7.37 (t, *J* = 7.1 Hz, 1H, 6-H); ^13^C NMR (151 MHz, DMSO-*d*_6_) δ: 164.86, 163.26, 142.80, 141.59, 137.31, 136.60, 134.00, 131.80, 129.56, 129.09, 128.84, 128.63, 128.37, 122.39, 122.07, 120.89, 120.40, 114.48, 112.79; HRMS Calcd for 423.1007 (C_25_H_15_ClN_4_O, [M + H]^+^), found 423.1007.

#### 3.1.5. General Synthetic Procedure for Ethyl Hydrazonoformate **37**

A mixture of 1-phenyl-β-carboline-3-carbohydrazide **1** (302 mg, 1 mmol) in triethyl orthoformate (5 mL) was refluxed overnight. Then, the formed solid was collected by filtration, washed with petroleum ether, dried and recrystallized from methanol to give compound 37.

*Ethyl 1-phenyl-β-carboline-3-carbonylhydrazonoformate* (**37**). Yield: 92%; ^1^H NMR (600 MHz, DMSO-*d*_6_) δ: 11.98 (s, 1H, 9-NH), 11.27 (s, 1H, -CONHN=), 8.90 (s, 1H, 4-H), 8.45 (d, *J* = 7.9 Hz, 1H, 5-H), 8.10 (d, *J* = 7.1 Hz, 2H, Ar-H), 7.71 (d, *J* = 8.2 Hz, 1H, 8-H), 7.67 (t, *J* = 7.6 Hz, 2H, Ar-H), 7.63–7.59 (m, 2H, 7-H, Ar-H), 7.34 (t, *J* = 7.5 Hz, 1H, 6-H), 7.16 (s, 1H), 4.28 (t, *J* = 7.1 Hz, 2H, OCH_2_CH_3_), 1.35 (t, *J* = 7.1 Hz, 3H, OCH_2_CH_3_); ^13^C NMR (151 MHz, DMSO-*d*_6_) δ: 159.49, 146.47, 141.63, 140.44, 138.71, 137.71, 134.34, 130.22, 129.27, 128.83, 128.36, 122.15, 121.17, 120.42, 113.39, 112.76, 67.36, 15.19. ESI-MS: *m*/*z* 359.17 [M + H]^+^.

### 3.2. Biology

#### 3.2.1. Cell Culture

*Spodoptera frugiperda* (Sf9) cells were grown in Grace′s insect medium supplemented 10% fetal bovine serum (FBS), 0.33% yeast extract and 0.33% lactalbumin hydrolysate and cultivated at 28 °C in a humidified incubator 2.5% CO_2_. Cells were seeded with 1 × 10^6^ cells mL^−1^ density for the following treatment.

#### 3.2.2. Anti-Proliferation Assay

3-(4,5-Dimethylthiazole-2yl)-2,5-diphenyl (MTT) assay was utilized to evaluate the bioactivity of β-carboline derivatives. Sf9 cells were subjected to treatment with various concentrations of β-carboline derivatives. After 24 h incubation, 10 μL freshly prepared MTT (5 mg mL^−1^) was added, followed by incubation for 4 h in darkness. The supernatants were carefully discarded and 150 μL of dimethyl sulfoxide (DMSO) was added to dissolve the formazan crystal. The absorbance was measured at 490 nm by a microplate reader (BioTek, Winooski, VT, USA). The cytotoxic effect was calculated by the following formula.

$$\mathrm{Cell}\;\mathrm{growth}\;\mathrm{inhibition}\;\mathrm{rate}\;(\%)=\frac{{\mathrm{OD}}_{\mathrm{control}}-{\mathrm{OD}}_{\mathrm{treatment}}}{{\mathrm{OD}}_{\mathrm{control}}}\times 100$$

#### 3.2.3. Cell Morphological Observation

Sf9 cells were treated with various concentrations of harmine, and compounds **5** and **37**. Morphological characteristics of Sf9 cells were recorded using inverted phase contrast microscope (IPCM) (Lecia, Tokyo, Japan).

#### 3.2.4. Acridine Orange (AO) Staining Analysis

Sf9 cells were subjected to treatment with various concentrations of harmine, and compounds **5** and **37**. After 24 h incubation, cells were collected and resuspended by PBS, followed by staining with 1 µM acridine orange and incubation for 15 min in darkness. The morphology of Sf9 cells was observed and photographed using fluorescence microscopy (Olympus BX51, Olympus, Japan).

#### 3.2.5. Apoptosis Rate Analysis

Apoptosis assay was performed using Annexin V/FITC Kit (Nanjing KeyGEN Biotech. Co. Ltd., Nanjing, China). Sf9 cells were subjected to treatment with 12.5 μg mL^−1^ harmine, and compounds **5** and **37**, respectively. After 24 h incubation, the cells were collected, dyed by Annexin V-FITC and PI, and incubated for 15 min in darkness. Subsequently, the samples were processed in a FACSCalibur (Becton Dickinson, Franklin Lakes, NJ, USA) and data were analyzed using ModFit LT software (Becton Dickinson, USA).

#### 3.2.6. DNA Ladder Assay

Genomic DNA of different treatment groups was isolated by TIANamp Genomic DNA Kit (TIANGEN) and was subjected to agarose gel electrophoresis.

#### 3.2.7. Sf-Caspase-1 Activity Assay

Sf9 cells were treated with 12.5 μg mL^−1^ harmine, and compounds **5** and **37**, respectively. After 24 h incubation, cells were collected and suspended in lysis buffer at 4 °C for 15 min. After centrifuged at 2000 rpm for 10 min at 4 °C, the supernatant was transferred to another tube and protein concentration was determined by Brandford method. The supernatant of lysate (50 µL) was added to 50 μL of Reaction Buffer and 5 μL caspase-3 substrate and incubated at 37 °C for 4 h in the darkness. The samples were measured at 405 nm on Microplate Reader (Biotex, Winooski, VT, USA).

#### 3.2.8. Cell Cycle Analysis

Sf9 cells were treated with 12.5 μg mL^−1^ harmine, and compounds **5** and **37**, respectively. After 24 h incubation, a suspension of cells was fixed in 70% ice-cold ethyl alcohol for more than 2 h, followed by labeling with propidium iodide (PI). The labeled cells were analyzed using a Flow Cytometer. The percentage of cells in the G1, S and G2 phases of the cell cycle were determined using the Flowjo 7.6.1 software (FlowJo, LLC, Ashland, OR, USA).

#### 3.2.9. Insect Growth Inhibition against Tobacco Cutworm (*Spodoptera litura*)

The insect growth inhibition of compounds **5** and **37** against *S. litura* were evaluated using our previously reported methods [34,35]. Firstly, the compounds were prepared to various concentrations by dissolving compounds in DMSO and adding distilled water. Leaf discs (5 cm in diameter) were cut from fresh taro leaves were dipped in the test solution for 5 s and then air dried. The leaf discs were placed in a petri dish lined with filter paper, and then 10 3rd-instar larvae were transferred to the petri dish. Two days after treatment, larvae were provided with fresh, untreated leaves and maintained under standard rearing conditions. Each treatment was performed 3 times. The surviving larvae were weighed using an electron scale accurate to 0.0001 g before and after the treatment at 2, 4, 6, 8, 10, 12 and 14 days. The larvae were continued to rear until all butterflies in the control treatment emerged. The pupation rate and mean pupae weight were calculated.

## 4. Conclusions

In conclusion, a series of novel β-carboline 1,3,4-oxadiazole derivatives were designed, synthesized and investigated for in vitro cytotoxic activity against Sf9 cells and growth inhibitory activity against *S. litura*. Bioassay results indicated that all of these compounds exhibited significant in vitro cytotoxic activity and were more potent than harmine. Notably, compounds **5** and **37** not only displayed superior cytotoxicity and were more potent than CPT, but also induced cell apoptosis and cell cycle arrest at S phase and stimulated Sf-caspase-1 activation in Sf9 cells, indicating that Sf-caspase-1 was involved in the apoptotic mechanism of compounds **5** and **37** in Sf9 cells. Furthermore, in vivo bioassay also demonstrated that compounds **5** and **37** could significantly inhibit larvae growth of *S. litura* by decreasing the weight of larvae and pupae. From the result of bioassay, we can conclude that compounds **5** and **37** could be used as potential lead compounds for the development of novel insect growth regulators. Further studies on whether growth inhibition of insects is related to cell apoptosis are in progress.

## Figures and Tables

**Table 1:** In vitro cytotoxicity of compound **2–37** against sf9 cell.

Compound	R_1_	X	R_2_	IC_50_ (μmol L^−1^)	95% Confidence Interval
**2**	CH_3_	=S		35.49	23.87–49.90
**3**	Ph	=S		45.44	28.69–70.64
**4**	4-CH_3_-Ph	=S		47.35	29.99–87.66
**5**	4-F-Ph	=S		16.25	0.11–61.37
**6**	4-Cl-Ph	=S		12.80	6.36–20.96
**7**	4-Br-Ph	=S		34.23	20.84–55.59
**8**	4-CF_3_-Ph	=S		17.58	9.67–31.89
**9**	4-OCH_3_-Ph	=S		10.39	8.09–12.95
**10**	2-Cl-Ph	=S		27.69	7.99–57.07
**11**	3-Cl-Ph	=S		13.83	1.08–42.37
**12**	3-F-Ph	=S		22.37	16.71–29.03
**13**	3-NO_2_-Ph	=S		63.97	32.36–134.99
**14**	3,4-di-OCH_3_-Ph	=S		64.86	22.05–135.19
**15**	3,4,5-tri-OCH_3_-Ph	=S		14.22	8.01–22.03
**16**	3,4-diF-Ph	=S		213.34	171.16–293.30
**17**	3,4-diCl-Ph	=S		98.74	48.67–316.92
**18**	3-F-4-OCH_3_-Ph	=S		25.79	10.89–42.80
**19**	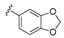	=S		56.61	34.02–90.47
**20**	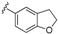	=S		19.11	14.48–25.90
**21**	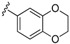	=S		30.84	3.78–70.23
**22**	2-naphthyl	=S		11.10	3.50–21.70
**23**	6-quinolyl	=S		197.81	130.28–380.25
**24**	CH_3_	=O		725.93	355.57–2562.85
**25**	Ph	=O		18.09	5.76–37.16
**26**	4-CF_3_-Ph	=O		27.35	10.22–88.94
**27**	3,4,5-tri-OCH_3_-Ph	=O		54.92	35.49–104.66
**28**	Ph		NH_2_	28.32	18.42–41.27
**29**	4-CF_3_-Ph		NH_2_	44.29	18.74–86.43
**30**			H	122.52	105.73–144.47
**31**			CH_3_	129.92	110.74–165.14
**32**			CH_2_CH_3_	66.63	45.36–97.30
**33**			cyclopropyl	127.76	102.16–167.65
**34**			Ph	144.66	87.87–463.36
**35**			4-CH_3_-Ph	>1000	-
**36**			4-Cl-Ph	>1000	-
**37**		-		3.93	1.03–8.01
Harmine				140.68	75.05–319.53
Camptothecin				18.95	12.43–25.68

## Supplementary Materials

The following are available online. Figure S1. Representative photographs of cell morphological change induced by compound **5** at various concentrations for 24 h, respectively. Figure S1. Agarose gel electrophoresis analysis of genomic DNA of cells treated by harmine at various concentrations.
