# Supplementary material for: Design, Synthesis and Bioactivity Evaluation of Novel β-carboline 1,3,4-oxadiazole Derivatives
Source: Molecules. 2017 Oct 29;22(11):1811. doi: 10.3390/molecules22111811 (PMC6150204; doi:10.3390/molecules22111811)
Supplement: Supplementary file 1 [file molecules-22-01811-s001.pdf]

Supplementary data

Design, synthesis, bioactivity evaluation and QSAR studies of novel β-carboline 1,3,4-oxadiazole derivatives

Zhi-Jun Zhang, Jing-Jing Zhang, Zhi-Yan Jiang, Guo-Hua Zhong*

Key Laboratory of Natural Pesticide and Chemical Biology, Ministry of Education, P.R. China, Lab of Insect Toxicology, South China Agricultural University, Guangzhou, 510642, P.R. China.

*Author to whom correspondence should be addressed. E-mail: guohuazhong@scau.edu.cn

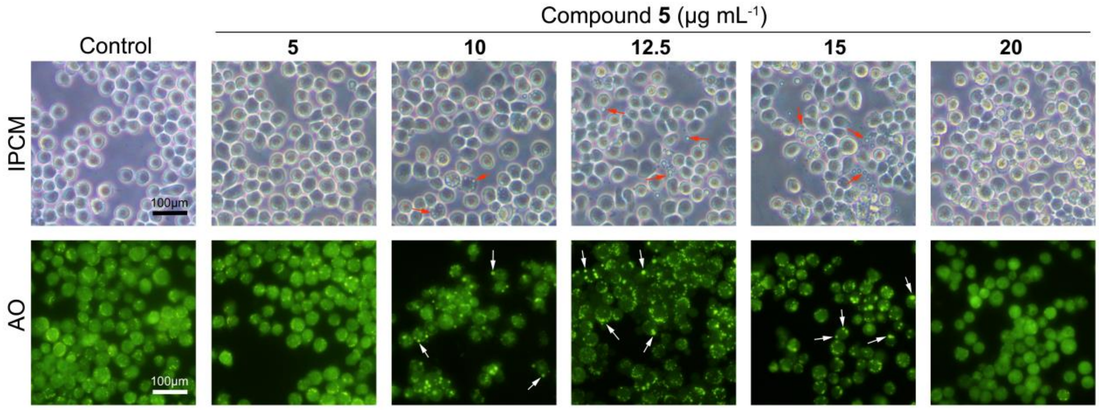

Figure S1. Representative photographs of cell morphological change induced by compound **5** at various concentrations for 24 h, respectively. IPCM: inverted phase contrast microscopy; AO: acridine orange. red and white arrow represented apoptosis bodies.

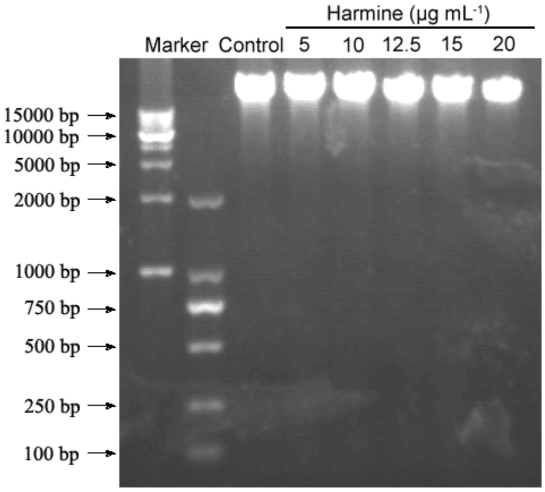

Figure S2. Agarose gel electrophoresis analysis of genomic DNA of cells treated by harmine at various concentrations.
